# Surgery for colorectal liver metastases: the impact of resection margins on recurrence and overall survival

**DOI:** 10.1186/1477-7819-12-127

**Published:** 2014-04-27

**Authors:** Jon-Helge Angelsen, Arild Horn, Geir Egil Eide, Asgaut Viste

**Affiliations:** 1 Department of Acute and Digestive Surgery, Haukeland University Hospital, N-5021 Bergen, Norway; 2 Department of Clinical Medicine, University of Bergen, N-5020 Bergen, Norway; 3 Department of Research and Development, Centre for Clinical Research, Haukeland University Hospital, N-5021 Bergen, Norway; 4 Department of Global Public Health and Primary Care, University of Bergen, N-5020 Bergen, Norway

**Keywords:** Colorectal liver metastases, Resection margin, Overall survival, Local recurrence, Time to recurrence, Preoperative chemotherapy

## Abstract

**Background:**

Several reports have presented conflicting results regarding the association between resection margins (RMs) and outcome after surgery for colorectal liver metastases (CLM), especially in the era of modern chemotherapy. The purpose of this study was to evaluate the impact of RMs on overall survival (OS), time to recurrence (TTR) and local recurrence (LR) status, particularly for patients treated with preoperative chemotherapy.

**Methods:**

A combined retrospective (1998 to 2008) and prospective (2008 to 2010) cohort study of consecutive patients with CLM without extrahepatic disease treated with primary resection at a medium volume centre.

**Results:**

A total of 253 patients with known R status and 242 patients with defined margin width were included in the study. Patients were stratified according to margin width; A: R1, <1 mm (n = 48, 19%), B: 1 to 4 mm (n = 77), C: 5 to 9 mm (n = 46) and D: ≥10 mm (n = 71). Median time to recurrence was 12.8 months, and after five years 21.5% had no recurrence. LR (inclusive combined recurrence in other hepatic sites or extrahepatic) occurred in 40 (16.5%) cases, most frequently seen with RMs below 5 mm. Five-year OS was 42.5% in R0 and 16.1% in R1 resections (*P* = 0.011). Patients were also stratified according to preoperative chemotherapy (n = 88), and the difference in five-year OS between R0 (45.1%) and R1 (14.7%) was maintained (*P* = 0.037). By multiple Cox regression analysis R1 resections tended to an adverse outcome (*P* = 0.067), also when adjusting for preoperative chemotherapy (*P* = 0.081).

**Conclusions:**

R1 resections for colorectal liver metastases predict adverse outcome. RMs below 5 mm increased the risk for LR and shortened the time to recurrence. Preoperative chemotherapy did not alter an adverse outcome in R1 vs. R0 patients.

## Background

Resection for colorectal liver metastases (CLM) has been well established during the last three decades, with a reported five-year survival of up to 64%, depending on selection criteria and preoperative risk factors [1-3]. In all intended curative cancer surgery a complete removal of the tumor is of major importance. During the 1980s and 1990s authors recommended ‘the 1 cm rule’ [4-7] that probably resulted in rejection of many patients from CLM surgery. Several reports from the last decade have shown that resection margins (RMs) are less important as long as R0 status is obtained [1,8-11]. In other reports 2 mm [12] and 5 mm [13] have been suggested as sufficient. Finally, some authors have even justified intended R1 resection following great progress in pre- and postoperative chemotherapy treatment due to an acceptable long-term outcome [14-16].

In an advanced stage IV cancer disease like CLM most patients are beyond curative treatment. In patients with resectable metastases, the surgical approach and the RMs are some of the few non-biological factors influenced by the surgeon. The purpose of this manuscript was therefore to analyse in detail the local recurrence (LR) pattern, time to recurrence (TTR) and overall survival (OS) with respect to the R1/R0 status and the magnitude of free RMs in patients with primary resection for CLM. We also wanted to explore whether chemotherapy altered the RMs impact on survival.

## Methods

Haukeland University Hospital is a tertiary referral centre located in Western Norway, and serves a population of one million. This study is a patient-based cohort with a consecutive series of patients with CLM treated at a single institution (1998 to 2010). Data from the period 1998 to 2008 were retrospectively recorded, and prospectively collected from 2008 to 2010. Data were retrieved from the patients’ medical records. All patients were prospectively followed up with respect to survival and other characteristics until November 2012. Variables analysed were TNM stage of primary tumour, time in months between resection of primary tumor and diagnosis of liver metastases (disease-free interval), number and size of metastases, chemotherapy (number of cycles, response and indication), date of liver resection, complications and in-hospital mortality, recurrence and death (perioperative, cancer-related and other causes). RM status was obtained from the microscopic measurements in the histological reports. RMs <1 mm were defined as positive (R1), in accordance with Pawlik *et al.*[9].

### Preoperative evaluation

The selection criteria for surgery in our centre included a sufficient remaining tumour-free liver volume (30%) with adequate blood perfusion and bile drainage, and absence of: a) non-resectable extrahepatic metastases, and/or b) no disseminated disease as evaluated preoperatively. Patients with extrahepatic disease and R2 resections were excluded from the current study. Preoperative investigations included computed tomography (CT) scan of the chest and abdomen/pelvis, and tumour marker analysis (CEA: carcinoembryonal antigen). In cases with an inconclusive CT scan, magnetic resonance imaging (MRI) of the liver, contrast-enhanced ultrasound and 18 F-fluorodeoxyglucose ^18^(FDG)-positron emission tomography (PET)/CT scan were performed. Each patient was discussed in a multidisciplinary team meeting with surgeons, oncologists and radiologists.

### Chemotherapy

Preoperative chemotherapy (n = 88) was given in a perioperative setting (n = 43) or as a downstaging procedure (n = 40) in patients with initially deemed unresectable disease. Five patients developed CLM during adjuvant treatment with chemotherapy after resection of stage III colon cancer. We evaluated the outcome of chemotherapy by the Response Evaluation Criteria in Solid Tumour (RECIST) version 1.1 [17]. The size of the metastases was measured on CT scan by dedicated radiologists. All patients in the perioperative group were offered the FOLFOX regimen (fluorouracil, leucovorin and oxaliplatin) with an intended six cycles before and after surgery. They were evaluated with CT scan after three and six cycles. The indication for perioperative chemotherapy has changed during the period. A total of 17 patients were enrolled in the European Organisation for Research and Treatment of Cancer (EORTC) multicentre study 40983 and randomised for surgery alone (n = 7), or surgery with perioperative chemotherapy (n = 10) in the period 2001 to 2004 [18]. After that, patients <76 years with Eastern Cooperative Oncology Group (ECOG) performance status 0 to 1 and no previous treatment with oxaliplatin had been offered perioperative chemotherapy. In the downstaging group, patients were treated with several different chemotherapy regimens as listed in Table 1. First-line treatment with the Nordic FLOX or FLIRI regimen was most commonly used, optionally in combination with EGFR (endothelial growth factor receptor) inhibitors or angiogenesis inhibitors.

**Table 1:** Clinical characteristics and administration of chemotherapy in 253 patients with primary resection for colorectal liver metastases

**Variable, *statistics***	**Estimate**
Age in years, *median (range)*	66.1 (22.8, 89.2)
Gender *male/female ratio*	133/120
Synchronous metastases^a^, *n (%)*	115 (45.5)
Disease-free interval^b^ in months*, median (range)*	4 (-14,131)
Resections, *n*	253
Hemihepatectomy/lobectomy, *n (%)*	117 (46.2)
Wedge/segment resections, *n (%)*	136 (53.8)
Simultaneous radiofrequency ablation, *n (%)*	12 (4.7)
Two-stage resections, *n (%)*	3 (1.2)
Simultaneous colorectal cancer surgery, *n (%)*	14 (5.5)
Extent of resection margin in mm, *median (range)*	4 (0-50)
Number of metastases, *median (range)*	2 (1, 12)
Metastases diameter in cm, *median (range)*	3.0 (0.2,15.0)
Bilobar metastases, *n (%)*	94 (37.2)
Number of resections/patient (1/2/3/4/5)	203/36/11/2/1
In-hospital mortality, *n (%)*	4 (1.6)
Follow-up survivors in years, *median (range)*	4.7 (1.9-12.9)
Chemotherapy preoperatively, *n (%)*	88 (34.8)
Downstaging, *n (%)*	40 (15.8)
Perioperative^c^, *n (%)*	43 (17.0)
Adjuvant after colon surgery, *n (%)*	5 (2.0)
Type of chemotherapy	
FOLFOX^d^, *n (%)*	71 (81.6)
FOLFIRI^e^/+bevacizumab, *n (%)*	8/3 (9.1/3.4)
FOLFIRI + cetuximab, *n (%)*	1 (1.1)
FLV^f^, *n (%)*	3 (3.4)
Other combinations, *n (%)*	5 (5.7)
Outcome of chemotherapy^g^ (RECIST)	
Partial response, *n (%)*	52 (59.1)
Stable disease, *n (%)*	32 (36.4)
Progression, *n (%)*	2 (2.3)
Unknown, *n (%)*	2 (2.3)
Number of cycles	
≤3	11 (12.5)
4-6	40 (45.4)
7-12	27 (30.7)
>12	7 (8.0)
Unknown	3 (3.4)
Chemotherapy adjuvant, *n (%)*	44 (17.4)
After neoadjuvant, *n (%)*	30 (11.9)
After downstaging, *n (%)*	7 (2.8)
Without preoperative chemotherapy, *n (%)*	7 (2.8)

^a^Synchronous metastases: detected <1 month after surgery of primary colorectal tumor; ^b^disease-free interval: time from resection of primary colorectal tumor to detection of hepatic metastases; ^c^all patients were offered the FOLFOX regimen; ^d^FOLFOX (oxaliplatin, 5-fluorouracil, leukovorin); ^e^FOLFIRI (irinotecan, 5-fluorouracil, leukovorin); ^f^FLV (5-fluorouracil, leukovorin); ^g^RECIST-criteria measured by computed tomography scan.

### Surgical procedures

Surgical techniques included subcostal incision, intraoperative ultrasonography, occasionally repeated inflow control (the Pringle manoeuvre), and transection using Ultracision, Kelly clamp and Cavitron Ultrasonic Surgical Aspirator (CUSA). Throughout the period we have intended to achieve a parenchyma-sparing approach, with wedge resections whenever possible. Formal resections (hemihepatectomies or lobectomies) have been reserved for metastases placed centrally or near the hepatic veins. To increase intended complete tumour eradication, intraoperative radiofrequency ablation, and portal vein ligations/embolization with two-stage resections have been performed. Simultaneous colorectal cancer surgery has been reserved for healthy patients with colon cancer and less advanced CLM. Further details are listed in Table 1.

### Surveillance

Follow-up after surgery included CT scan of the chest, abdomen and pelvis every three months for the first two years, and thereafter every six months for the next three years. Serum level of CEA tumour marker was obtained every third month. We defined LR by CT scan as a new lesion in contact with the previous resection surface. The resected area was easily detected with CT due to the wide use of metallic clips during the transection. The data were based on the first detection of recurrence. Data of recurrence were not available in four patients. Patterns of recurrence were stratified according to LR, hepatic recurrence (without LR) and extrahepatic recurrence. LR included patients with a) LR only, b) LR and relapse in other sites of the liver and c) LR with concomitant new extrahepatic lesions. During the follow-up, thirteen patients died from causes other than colorectal cancer, and six from treatment-related causes. These patients were also included in the analysis of OS, according to the definition stated by Punt *et al.*[19].

### Statistical analysis

Variables with possible impact on OS like RM, age, size, number of metastases, bilobar distribution, disease-free interval and TNM stage of primary tumour were analysed with univariate and multivariate survival methods. The exact chi-square (*χ*^2^) test was used for categorical variables, the *t* test for normally distributed variables, and the Mann–Whitney *U* (MWU) test for non-normally distributed continuous variables. The Kruskal-Wallis one-way analysis of variance test was used to compare more than two non-normally distributed samples. Multinomial logistic regression was used to evaluate LR in relation to RMs. Survival was estimated by the Kaplan-Meier method [20] and tested for significance with the log-rank test [21]. Multivariate analysis was performed as Cox proportional regression [22]. Continuous predictors such as RMs were also modelled using multiple fractional polynomial regressions [23]. A *P* value ≤0.05 was considered significant. OS was defined as time from resection to death irrespective of cause, and TTR was defined as the interval between resection and the detection of a local or distant relapse [19]. All analyses were performed using SPSS Statistics version 19 (IBM Corp., Armonk, NY, USA) and Stata 12 statistical software (StataCorp, College Station, TX, USA). We decided to use TTR rather than disease-free survival as a parameter in assessing recurrence patterns, since the latter has treatment-related and non-cancer-related deaths as endpoints, which could be misleading according to the definition by Punt *et al.*[19].

### Ethics

The Regional Committee of Ethics of Western Norway Health Authority approved the study, with an exemption to the requirement for obtaining informed consent from patients included in the retrospective part (1998 to 2008). In the prospective part (2008 to 2010) patients were enrolled through written consent.

## Results

In total, 278 patients underwent 353 resections in the 13-year period. Among these, 270 patients underwent a primary (first) liver resection. Eight patients were admitted from other hospitals for re-resections. Fourteen patients (5.2%) with primary resectable extrahepatic metastases (thirteen pulmonary and one pelvic) were not included in the current study. One patient could not complete the second procedure of a two-stage liver resection due to progression of disease. The R0/R1 status was not obtained in two patients, whereas the exact resection margin (in millimetres) could not be defined in eleven cases.

Finally, a total of 253 patients with known R status and 242 patients with a defined margin width were eligible for further analysis. Patients were further sub-grouped according to margin width obtained from the histological report; A: R1, <1 mm (n = 48), B: 1 to 4 mm (n = 77), C: 5 to 9 mm (n = 46) and D: ≥10 mm (n = 71). Clinical and pathological features are listed in Table 1. Positive microscopic margins (R1) were found in 48 cases (19.0%).

### Patient and tumour demographics

R1 patients had more advanced disease compared to R0 according to bilobar locations (*P* = 0.007, *χ*^2^-test) and number of metastases (*P* = 0.099, MWU test). There was no significant difference between R0 and R1 patients in the TNM status of primary tumour in colon or rectum, American Society of Anesthesiologists (ASA) score, size of the metastases and the use of preoperative chemotherapy. Postoperative chemotherapy was administered more frequently in R1 patients (n = 12 of 48, 25.0%) compared to R0 (n = 31 of 205, 15.1%), *P* = 0.016, *χ*^2^-test. In the chemotherapy group, there was no difference in number of R1 resections between patients with partial response or stable disease using the RECIST criteria (*P* = 0.575, *χ*^2^-test). In the perioperative and the downstaging group a total of thirty (69.8%) and seven (17.5%) patients, respectively, underwent postoperative chemotherapy (*P* <0.0001, *χ*^2^-test).

### Patterns of recurrence

**Figure 1:**
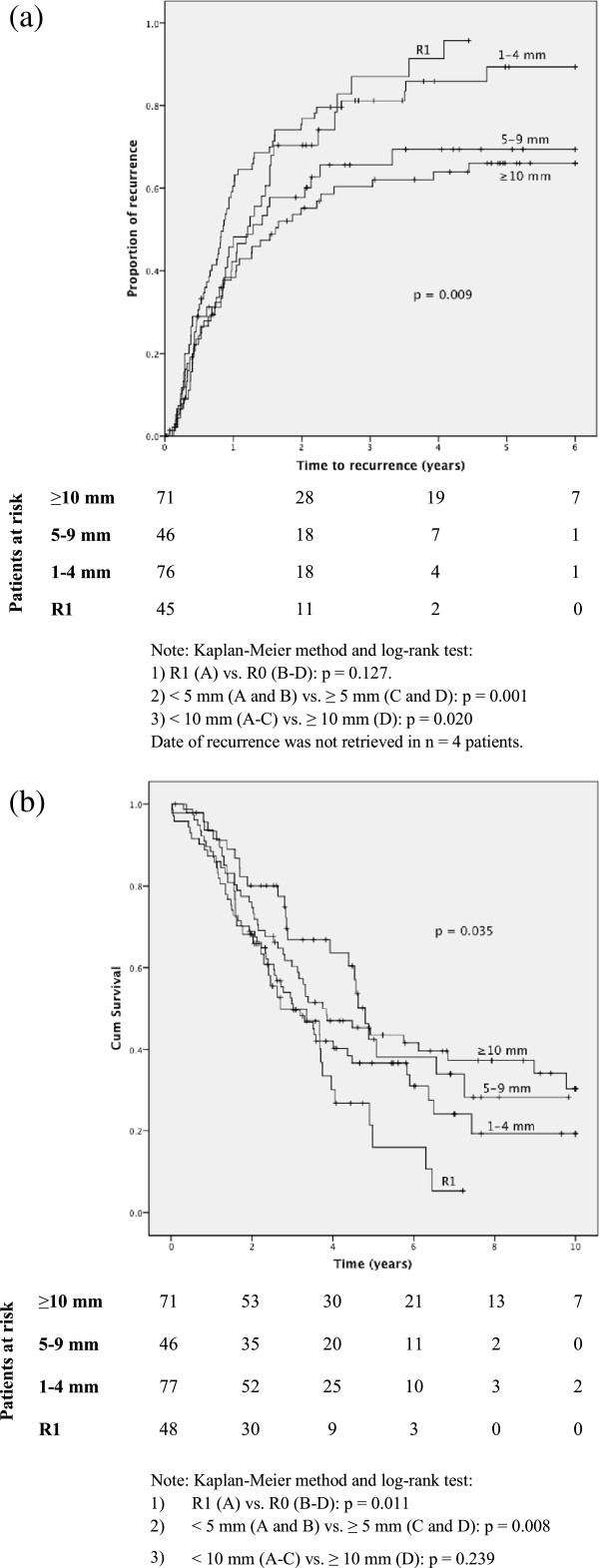
Time to recurrence (a) and overall survival (b) according to resection margins in n = 242 patients with primary resection for colorectal liver metastases.

Global recurrent disease occurred in n = 175 (72.3%) patients, whereas involvement of the resection surface was found in 40 cases (16.5%). Further details are listed in Table 2. We found a lower global recurrence in the groups C and D compared to A and B. The risk for recurrence according to RMs (A to D) was assessed with a multinomial logistic regression, as detailed in Table 3. The odds ratios for LR were significantly higher in groups A and B relative to group D. RMs did not seem to impact hepatic recurrence, whereas extrahepatic recurrence was more frequent compared to no recurrence with RMs <5 mm (0.005). A total of 21.5% of the patients were recurrence-free after five years. TTR increased significantly (*P* = 0.009) with the increasing extent of the RMs (Figure 1a), but this difference was repealed when we omitted those patients (n = 40) with all kinds of LR (*P* = 0.097). We also detected a non-significant difference in five-year TTR between R0 (24.5%) and R1 (0%, *P* = 0.127). No additional benefit for TTR was seen with RMs beyond 10 mm, where the groups C and D were nearly equal in outcome (Figure 1a).

**Table 2:** **Global recurrence and local recurrence (LR) following n** = **242 primary resections for colorectal liver metastases according to resection margins (RMs)**

	**Resection margins**				
**Recurrence**	**A (R1) n (%)**	**B (1-4 mm) n (%)**	**C (5-9 mm) n (%)**	**D (≥10 mm) n (%)**	**All n (%)**
LR only	7 (14.6)	3 (3.9)	3 (6.5)	1 (1.3)	14 (5.8)
LR and hepatic	3 (6.3)	3 (3.9)	3 (6.5)	1 (1.3)	10 (4.1)
LR and extrahepatic	6 (12.5)	10 (13.0)	0 (0.0)	0 (0.0)	16 (6.6)
LR (total)^1^	16 (33.3)	16 (20.7)	6 (13.0)	2 (2.8)	40 (16.5)
Hepatic only	8 (16.7)	15 (19.5)	7 (15.2)	22 (31.0)	52 (21.5)
Extrahepatic	13 (27.1)	31 (40.3)	17 (37.0)	18 (25.4)	79 (32.6)
Unknown	2 (4.2)	1 (1.3)	0 (0.0)	1 (1.3)	4 (1.7)
Global^2^	39 (81.3)	63 (81.8)	30 (65.2)	43 (60.5)	175 (72.3)
No recurrence	9 (18.8)	14 (18.2)	16 (34.8)	28 (39.4)	67 (27.7)
Total	48 (100)	77 (100)	46 (100)	71 (100)	242 (100)

Extrahepatic recurrence: recurrence outside the liver with or without hepatic involvement. Statistics: *P* = 0.0003 (*χ*^2^ two-sided exact test) when all groups were included.

^1^LR (total): the sum of ‘LR only’, ‘LR and hepatic’ and ‘LR and extrahepatic’.

^2^Global recurrence: the sum of ‘LR (total)’, ‘hepatic only’, ‘extrahepatic’, and ‘unknown recurrence’.

**Table 3:** **Results from multinomial logistic regression of recurrence according to resection margins (RMs) in n** = **242 patients with known recurrence status after primary resection for colorectal liver metastases**

**Recurrence**^**†**^	**RM**	**OR**	**95% CI**	***P *** **value**
Local	R1, <1 mm	24.89	(4.77, 129.69)	0.0001
(n = 40)	1-4 mm	16.00	(3,22, 79.56)	0.001
	5-9 mm	5.25	(0.95, 29.15)	0.058
	≥10 mm	1.00	Reference	
Hepatic only	R1, <1 mm	1.13	(0.38, 3.42)	0.827
(n = 52)	1-4 mm	1.36	(0.55, 3.41)	0.508
	5-9 mm	0.56	(0.20, 1.59)	0.274
	≥10 mm	1.00	Reference	
Extrahepatic*	R1, <1 mm	2.25	(0.80, 6.33)	0.126
(n = 79)	1-4 mm	3.44	(1.45, 8.18)	0.005
	5-9 mm	1.36	(0.67, 4.08)	0.276
	≥10 mm	1.00	Reference	

^†^Reference category is ‘no recurrence’ (n = 67). Unknown recurrence pattern in n = 4 patients. *Extrahepatic: recurrence outside the liver with or without hepatic involvement. RM*,* resection margins; OR*,* odds ratio; CI*,* confidence interval.

A total of 50 of 253 (19.8%) patients underwent a second operation for resectable recurrence. Twenty (40.0%) of these were due to LR after the first resection. In 27 cases (54.0%), recurrence was found in other sites of the liver whereas only three patients (6.0%) had combined intra- and extrahepatic relapses. In patients with LR only, 11 of 14 (78.6%) patients were resected. Of the 48 patients having a primary R1 resection, 15 (31.3%) underwent a second operative procedure.

### Overall survival

Five- and ten-year OS survival rates were 38.7% and 23.0%, respectively, whereas median OS was 45.0 months. Five-year OS of R0 vs. R1 was 42.5% and 16.1%, (*P* = 0.011, Figure 1b), whereas median OS in R0 and R1 were 48.1 and 32.4 months, respectively. By sub-grouping according to margin width (A to D), an increased OS was seen in the univariate analysis (*P* = 0.035, see Figure 1b). However, there was no extra benefit when the RMs exceeded 10 mm (group C vs. D). Patients were also stratified according to preoperative chemotherapy (n = 88), and the difference in five-year OS between R0 and R1 was maintained (*P* = 0.037). In the perioperative group (n = 43), a non-significant difference (*P* = 0.502) in five-year OS was seen between R1 (34.3%) and R0 (54.2%). In the downstaging group (n = 40), the five-year OS was 40.2% for the R0 cases vs. none survivors in the R1 group (*P* = 0.017). Patients with initially unresectable metastases had more extensive disease evaluated as the average number of metastases (3.6) compared to the perioperative (2.5) and the surgery alone group (2.3), using Kruskal-Wallis test (*P* = 0.002). Positive RMs also predicted a borderline significant adverse outcome in the Cox proportional hazards model (*P* = 0.067), along with age, ASA score, number of metastases, size of the metastases and repeated resections (Table 4). When using the RM sub-groups (A to D) in the Cox model (*P* = 0.111) and the RMs as a continuous variable (*P* = 0.099), significance was not reached. We neither found any substantial differences in OS with a cut-off margin of 5 mm (*P* = 0.194). We also applied multiple fractional polynomials in the Cox regression model without identifying any non-linear relationships between RMs and OS. When adjusting for patients offered preoperative chemotherapy, multivariate analyses revealed RMs still to be a borderline significant factor predicting adverse OS (*P* = 0.081). However, in contrast to the rest, there was an adverse effect on OS of R1 vs. R0 in the downstaging group (test of interaction *P* = 0.020), adjusting for the same variables as listed in Table 4. No such effect was evident in the perioperative group.

**Table 4:** Results from Cox regression analysis of resection margins and other factors affecting overall survival in 253 patients after primary resection for colorectal liver metastases

		**Overall survival**					
		**Univariate**			**Multivariate**		
**Variable**	**n**	**HR**	**95% CI**	***P***	**HR**	**95% CI**	***P***
Age/10 y	253	1.32	(1.12, 1.54)	<0.001	1.29	(1.08, 1.54)	0.005
DFI, months	253	0.91	(0.80, 1,03)	0.122	0.91	(0.79, 1.05)	0.187
Number of metastases	253	1.19	(1.10, 1.28)	<0.001	1.31	(1.17, 1.45)	<0.001
Metastasis diam, cm	242	1.11	(1.03, 1.19)	0.006	1.09	(1.02, 1.18)	0.023
RM status R0	205	1.00	Reference		1.00	Reference	
R1	48	1.69	(1.14, 2.51)	0.014	1.53	(0.98, 2.39)	0.067
T stage				0.931			0.897
T2	25	1.00	Reference		1.00	Reference	
T3	180	1.10	(0.65, 1.87)		0.99	(0.54, 1.81)	
T4	34	1.11	(0.57, 2.19)		0.88	(0.40, 1.90)	
N stage				0.506			0.229
N0	91	1.00	Reference		1.00	Reference	
N1	103	1.19	(0.83, 1.72)		1.32	(0.88, 1.98)	
N2	51	1.27	(0.81, 1.98)		1.49	(0.92, 2.42)	
ASA score	253	1.62	(1.20, 2.19)	0.002	1.61	(1.13, 2.29)	0.009
Bilobar No	159	1.00	Reference		1.00	Reference	
Yes	94	1.38	(1.01, 1.89)	0.048	1.22	(0.80, 1.86)	0.353
Re-resections		0.78	(0.60, 1.01)	0.045	0.71	(0.52, 0.97)	0.016

HR*,* hazard ratio; CI*,* confidence interval; DFI*,* disease-free interval (time between resection of the primary tumor in the colon or rectum and the detection of hepatic metastases); RM*,* resection margin; T and N stage, analysis of primary tumor in colon or rectum; ASA, American Society of Anesthesiologists. Unknown T-stage n = 14, unknown N-stage: n = 8, unknown diameter of metastases: n =11.

Finally, we conducted survival calculations according to the site of recurrence and the involvement of LR independently of RMs. We could not reveal any difference in five-year OS between LR (total), hepatic-only and extrahepatic recurrence (*P* = 0.947). Patients with LR only proved a better five-year OS compared to patients with recurrence at other sites (35.9% vs. 25.4%, *P* = 0.048). Within the latter group we neither found any substantial differences in OS (*P* = 0.130).

## Discussion

The main finding in this study was that positive RMs influenced overall survival after resection for CLM. LR occurred more frequently and TTR was shorter in RMs <5 mm. Following preoperative chemotherapy, negative margins were still a prerequisite for achieving an improved survival.

Our study demonstrated that positive margins were related to a more dismal prognosis. This is consistent with the majority of other comparable reports [1,9,24-26]. Even with a consensus on obtaining free margins after liver resections there are still conflicting results about the sufficient magnitude of the RMs and its impact on recurrence and survival. Several studies have shown that local recurrence and survival were independent of the extent of the free margins [8,9,11,27]. In addition to the benefit of R0, we found an increasing OS and TTR in patients with RMs >5 mm (Figures 1a and b). No additional advantage was found for free RMs beyond this limit. In the report from Nuzzo *et al.* a RM ≤5 mm was associated with a greater risk of LR, as well as reduced disease-free survival (DFS) and OS [13]. Likewise, Vandeweyer *et al.* demonstrated that a RM >1 mm improved OS. However, a margin beyond 1 mm did not yield any detectable advantage in survival [28]. In a large series of 2,715 prospective collected patients Hamady *et al.* stated that 1 mm free margin was sufficient to obtain a five-year DFS of 33%. An extra margin width did not provide DFS advantage in this study [29]. Konopke *et al.* showed that even though the size of the RMs did not affect overall survival, a resection margin below 3 mm increased hepatic and overall recurrence [30]. Wray *et al.* found that RM <1 cm was a powerful factor in increasing the risk for local and distant recurrence as well as DFS [31]. The result was, however, not confirmed in a multivariate setting when only R0 cases were included.

Several studies have through genetic techniques detected tumour DNA up to 4 mm from the tumour border, and thereby determining a rational basis for the extent of surgical excision [12,32-34]. We also demonstrated that RMs plays a key role in the development of LR independently of recurrence in other sites of the liver and/or extrahepatic (Tables 2 and 3) using multinomic logistic regression. Furthermore, no correlation was detected between RMs and intra- or extrahepatic relapse without LR involvement (Table 3). Surprisingly, we detected an increased risk for extrahepatic recurrence in patients with less than 5 mm free margins. We have no plausible explanation for this finding, and the results may suggest that RMs might be surrogates of the extent of the disease. This is also visualized through a fairly high level of recurrence (89.4%) in the group B (1 to 4 mm, Figure 1), as 40.3% of these patients had extrahepatic recurrence (Table 2). We hypothesise that intra- or extrahepatic relapse (without LR involvement) is based on progression of preoperatively non-detectable micro-metastases and not the impact of RMs. Unlike our report, de Haas *et al.* found that R1 was associated with intrahepatic recurrence, whereas no difference in surgical margin recurrence was seen between R0 and R1 [14]. Likewise, in the multi-institutional study of 1,669 patients by de Jong *et al.*, R1 resection was associated with intrahepatic recurrence, whereas extrahepatic disease developed independently of margin status [3].

In the study by Are *et al.* the RMs were analysed as a continuous variable [35]. They found no difference in survival between positive margins and sub-centimetre resections (*P* = 0.31) in the multivariate analysis, whereas patients with RM >1 cm had a significantly improved outcome. Nevertheless, the authors observed a favourable survival in sub-centimetre R0 resections, and they concluded that these patients should not be denied hepatic resections.

In some published articles, with initially marginally or non-resectable CLM receiving preoperative chemotherapy, the important role of free margins were found to be less important [14-16]. In the current study, we found an improved OS for R0 vs. R1 in patients receiving preoperative chemotherapy. Our data indicates that R0 resections should be strived for in these patients. This finding also corresponds with recently published studies [36,37]. In patients with initially unresectable metastases successfully treated with chemotherapy, positive margins predicted in the univariate model an adverse outcome (*P* = 0.017), but this finding was not evident in resectable patients offered perioperative chemotherapy (*P* = 0.502). In the multivariate analysis this difference was confirmed. In the first group, postoperative chemotherapy was administered more rarely (17.5%) compared with the latter group (69.8%). In other settings like stage III colon cancer, adjuvant chemotherapy regimens have proved to expose and reduce the recurrence rates [38,39]. We hypothesise the same mechanism in R1 patients, where adjuvant chemotherapy may suppress any remaining metastatic disease, leading to an increase in TTR and OS. This resembles the trial by Tranchart *et al.*[37].

An exact measurement of RMs is impeded by the application of surgical devices such as the ultrasonic aspirator, harmonic scalpel, and Kelly clamp-crushing technique, which removes a small rim of liver tissue during the transection. An overestimation of R1 cases might be the consequence [9,14]. Likewise, the invasive irregular growth pattern in liver metastases, combined with a rough transection surface, makes the histological examination less reliable in narrow margins. The increasing use of chemotherapy may also complicate the measurement of RMs due to a more irregular surface, as reported by Ng *et al.*[34].

Several studies have demonstrated an effect of R1 resections on OS in univariate analyses, but have not confirmed this finding in a multivariate setting [9,13,35]. This result has led to a discussion whether R status is a surrogate of other biologic factors such as size, number, growth patterns and distribution of the metastases, rather than an independent predictor for adverse outcome. In the current trial R1 was of borderline significance in the multivariate analysis (*P* = 0.067). However, a more advanced disease in patients undergoing R1 vs. R0 resection is reflected by a higher incidence of bilobar distribution and number of metastases. This is consistent with other recognized reports [9,14,35]. The advancement of disease reflected in number and size of metastases appears to have greater impact on survival than RMs. Based on our findings we advocate that R0 should be performed despite no clear significance in the Cox model. We also assume with a larger number of patients in the cohort, the significance might be obtained.

We reported a rather high incidence of LR (total) and global recurrence of 40 (16.5%), and 175 (72.3%) patients, respectively, which is somewhat higher than other studies [9,12,13]. The RMs (groups A to D) did not influence the TTR when patients with LR (total) were excluded from the analysis (*P* = 0.097). A similar finding in TTR was evident between R1/R0 (*P* = 0.403). However, we could neither detect any worse OS in patients with LR (total) compared with patients with recurrence at other sites. A fairly high proportion of patients with LR were offered repeated resections with curative intent. In patients with LR only, 78.6% underwent a second resection, following better OS compared with recurrence in other sites. We could not obtain a different OS among patients with relapse in other localisations. Despite a high recurrence rate, we have obtained a five-year OS of nearly 40% and a median OS of 45 months. We assume an aggressive multimodal treatment and with repeated resections in patients with advanced disease and marginally resectable metastases may be justified despite the high number of relapse [14]. Based on this, patients with suspected narrow RMs should not be excluded from resection for colorectal liver metastases.

## Conclusions

A positive resection margin predicted adverse OS after resection for colorectal liver metastases. Likewise, local recurrence and time to recurrence were influenced by positive margins. In addition, an increasing survival rate, a reduced recurrence (local and global) rate and a longer time to recurrence were seen in patients with RM >5 mm, but could not be verified beyond this extent. In an era with expanding use of chemotherapy, our study supports that R0 resections are still important in order to obtain the best outcome in patients treated with resection for colorectal liver metastases.

## Abbreviations

CEA: carcinoembryonal antigen; CLM: colorectal liver metastases; CT: computed tomography; DFS: disease-free survival; LR: local recurrence; OS: overall survival; RM: resection margin; TTR: time to recurrence.

## Competing interests

The authors declare that they have no competing interests. There was no grant support for this study.

## Authors’ contributions

All the authors have fulfilled the ICMJE guidelines, according substantial contribution to this study. JHA has been in charge of the data collection. All the authors have participated in the design, acquisition, data analysis and interpretations. GEE is a medical statistician, and has been in charge of the statistical calculations and interpretations of the collected data. All the authors have contributed in the drafting and have revised the manuscript critically before submission. They have all given their final approval of this version to be published, and take full responsibility for all the aspects and results in this manuscript.
